# Development of collagenous scaffolds for wound healing: characterization and in vivo analysis

**DOI:** 10.1007/s10856-023-06774-8

**Published:** 2024-02-05

**Authors:** Jéssica Peixoto Rodrigues, Jéssica Regina da Costa Silva, Bruno Antônio Ferreira, Lucas Ian Veloso, Ludmila Sousa Quirino, Roberta Rezende Rosa, Matheus Carvalho Barbosa, Cláudia Mendonça Rodrigues, Paula Batista Fernandes Gaspari, Marcelo Emílio Beletti, Luiz Ricardo Goulart, Natássia Caroline Resende Corrêa

**Affiliations:** 1 Laboratory of Nanobiotechnology, Institute of Biotechnology, Federal University of Uberlandia, Av. Amazonas s/n, Campus Umuarama BL-2E, SL-248, Uberlândia, Minas Gerais 38400-902 Brazil; 2 Department of Physiological Sciences, Federal University of Uberlândia, UFU, Uberlândia, MG Brazil; 3 Department of Cell Biology, Histology and Embryology, Federal University of Uberlândia, UFU, Uberlândia, MG Brazil

## Abstract

The development of wound dressings from biomaterials has been the subject of research due to their unique structural and functional characteristics. Proteins from animal origin, such as collagen and chitosan, act as promising materials for applications in injuries and chronic wounds, functioning as a repairing agent. This study aims to evaluate in vitro effects of scaffolds with different formulations containing bioactive compounds such as collagen, chitosan, N-acetylcysteine (NAC) and ε-poly-lysine (ε-PL). We manufactured a scaffold made of a collagen hydrogel bioconjugated with chitosan by crosslinking and addition of NAC and ε-PL. Cell viability was verified by resazurin and live/dead assays and the ultrastructure of biomaterials was evaluated by SEM. Antimicrobial sensitivity was assessed by antibiogram. The healing potential of the biomaterial was evaluated in vivo, in a model of healing of excisional wounds in mice. On the 7th day after the injury, the wounds and surrounding skin were processed for evaluation of biochemical and histological parameters associated with the inflammatory process. The results showed great cell viability and increase in porosity after crosslinking while antimicrobial action was observed in scaffolds containing NAC and ε-PL. Chitosan scaffolds bioconjugated with NAC/ε-PL showed improvement in tissue healing, with reduced lesion size and reduced inflammation. It is concluded that scaffolds crosslinked with chitosan-NAC-ε-PL have the desirable characteristics for tissue repair at low cost and could be considered promising biomaterials in the practice of regenerative medicine.

**Graphical Abstract:**

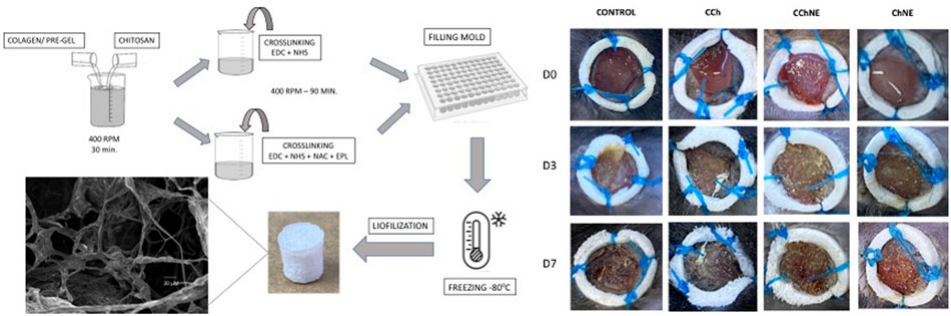

## Introduction

The skin is the human body’s main protective barrier from pathogens. In cases of dermis injury, this barrier is impaired. To prevent possible contamination, dehydration, heat loss and other damage, it is necessary to promote rapid wound closure and regeneration of damaged skin to restore the barrier function. Effective repair requires communication and interaction between different types of cells, a process that is regulated at various levels [1–3].

The development of wound dressings from biomaterials has been the subject of research due to their unique structural and functional characteristics. Animal-derived hydrogels made of collagen and chitosan act as promising materials for applications in injuries and chronic wounds, working as a repair agent that guides the patient’s own skin cells to a compatible and organized matrix. Hydrogel-based dressings and matrices have the potential to satisfy requirements of an ideal dressing as they provide a consistent and conducive environment for healing wounds with an acceptable cosmetic appearance [4, 5].

Collagen-derived sponge dressings provide a unique set of properties necessary for the wound healing process, such as high water affinity, chemotactic property, absorption capacity, platelet activation and biocompatibility [4–6]. Furthermore, collagen can also be combined with other materials and additives to obtain synergistic effects, enhancing its therapeutic role in the treatment of chronic or infected wounds [5, 6].

Another compound, chitosan is a natural linear biopolymer composed of a hydrophilic surface that promotes cell adhesion, proliferation and differentiation, which gives it properties including biocompatibility, bioactivity, biodegradability and high viability in different shapes and structures [7, 8]. Furthermore, due to the positively charged amino groups in the structure of this carbohydrate, it becomes mucoadhesive, which promotes the ability to bind cell membranes. Not only does chitosan have adequate porosity, but several studies have proven that its combination with collagen has resulted in better performance in terms of regenerative capacity [7, 9]

In addition, ε-Poly-L-lysine (ε-PL) is a cationic peptide consisting of 25 to 35 L-lysine residues [10]. The ε-PL was discovered as an extracellular material produced by filamentous actinomycetes such as *Streptomyces albulus* and *Lysinopolymerus*, and has been used as a natural antimicrobial peptide that acts by inhibiting various microorganisms such as bacteria, yeasts and viruses [11–13]. Due to its biodegradation and biocompatibility properties, ε-PL has been widely used as a food preservative in industry [14].

Furthermore, N-acetylcysteine (NAC), the acetylated variant of the amino acid L-cysteine, is a source of sulfhydryl (SH) groups and is converted in the body into metabolites capable of stimulating glutathione (GSH) synthesis, promoting detoxification and acting directly as free radical scavengers. NAC has been used in clinical practice as a mucolytic agent in respiratory diseases; however, it also appears to have beneficial effects in conditions of oxidative stress, such as HIV infection, cancer, heart disease and smoking [15]. There is urging interest in the effects of NAC against oxidative stress associated with its antioxidant properties due to its rapid reaction with free radicals and reduced glutathione restitution as demonstrated by Douhib et al. [16] and Adil et al. [17].

Due to the various features of the abovementioned compounds, our group believes that a synergy between these components could potentialize a wound dressing made of them. Therefore, this study aimed to evaluate the in vitro and in vivo effects of scaffolds derived from collagen-rich biomaterial in different formulations containing bioactive compounds such as chitosan, NAC and ε-PL.

## Materials and methods

### Ethics statement

All of the experimental procedures involving animals were approved by the Institutional Research Board for Ethics on Animal Use (Comitê de Ética no Uso de Animais, CEUA) of the Federal University of Uberlândia (UFU-Brazil) under approval protocol number 094/19 (CEUA/UFU). All experiments were conducted according to the guidelines for the care and use of laboratory animals of the CEUA/UFU.

### Preparation of collagen-based scaffolds with different concentrations

Chitosan (Sigma-Aldrich, C3646) was diluted in 0.1 M acetic acid (Dinamica) at the concentrations of 1, 1.5, 2 and 3 mg/mL. For solubilization, it was kept under agitation (400 rpm) in an IKA RT15 Magnetic Stirrer, overnight at room temperature. Commercial collagen (Sigma-Aldrich, C2124-50ML, 6 mg/mL, on average) was diluted in 0.01 M HCl (Dinamica) at concentrations of 1, 1.5, 2 and 3 mg/mL.

For the development and evaluation of the scaffold, 6 different formulations were performed at the concentrations of 1, 1.5, 2 and 3 mg/mL for comparison: Collagen - NAC - ε-PL (CNE), Collagen - Chitosan (CCh), Collagen - Chitosan - NAC - ε-PL (CChNE), Chitosan - NAC - ε-PL (ChNE). As controls, chitosan (Ch) and collagen (C) solutions were used.

To prepare the CNE and QNE scaffolds, 1 mg/mL of NAC (Sigma-Aldrich, A7250-1kG, >99%) and ε-PL (BioSynth, MW 3500–4500 Da, >98%) was added to the collagen and chitosan solutions previously diluted in concentrations of 1, 1.5, 2 and 3 mg/ml and homogenized.

To prepare the CCh scaffolds, chitosan was added in a 1:1 ratio to the previously diluted collagen according to the protocol adapted from Deng et al. [18]; and the result was kept under agitation (400 rpm) at room temperature for 20–30 min. Subsequently, 1 mg/mL of 1-Ethyl-3-(3-dimethylaminopropyl) carbodiimide - EDC (Sigma-Alcrich, 03449-1 G) and 1 mg/mL of N-Hydroxysuccinimide - NHS (Sigma-Alcrich, 56485-250MG) was added for crosslinking chitosan with collagen adapted from the method of Cao et al. [19] and kept under agitation (400 rpm) at RT for 90–120 min.

After the crosslinking of collagen and chitosan, 1 mg/mL of NAC and ε-PL was added to the solutions for preparing the CChNE scaffolds. For molding the scaffolds, each formulation and its respective control were plated at a volume of 200 to 300 µL per well (96-well plate) and frozen at −80 °C for 1 h. Then they were lyophilized overnight.

### Cell viability assay

To assess cytotoxicity, resazurin was used. Due to the characteristics of the scaffold that becomes solubilized in the culture medium, the indirect contact method by component extraction was chosen. Human keratinocytes cells (HaCat) were maintained in Dulbecco’s Modified Eagle’s Medium (Gibco), supplemented with 10% fetal bovine serum (Cultilab) and 1% antibiotic/anticyotic solution (Gibco) under standard culture conditions (37 °C, 95% humidified air, and 5% CO_2_) until confluence. For this assay, cells were plated in a 96-well plate in the amount of 1 × 10^4^ per well and kept in a CO_2_ oven for 24 h at 37 °C.

The scaffolds were prepared at different concentrations (1, 1.5, 2 and 3 mg/mL) in triplicate and incubated in 200 µL of culture medium per scaffold and kept at 37 °C for 24 h. After incubation, 100 µL of the medium containing scaffold extract was transferred to a 96-well plate containing 1 × 10^4^ cells per well. For positive control, 5% Dimethylsulfoxide - DMSO (Sigma-Aldrich) was added. After 72 h, cells were incubated with 20 µL/well of 3 nM Resazurin solution (Sigma-Aldrich) and maintained for 3 h at 37 °C. Cell viability was performed based on the mean fluorescence (Fl):

$$Viability\,( \% )=Fl(treated)\ast 100/Fl(control)$$

According to the results of this screening, the non-cytotoxic dose for the cell was chosen to proceed with the other assays.

### Live-dead assay

The scaffolds were prepared at the chosen concentration, incubated in 200 µL of culture medium per well and kept in an incubator at 37 °C for 24 h. After incubation, 100 µL of scaffold extract was collected and transferred to a 96-well plate containing 1 × 10^4^ cells per well. For positive control 5% DMSO was added. After 72 h, the medium was removed and, in each well, 100 µL of 3 µM calcein AM (Invitrogen – Thermo Fisher Scientific) and 2.5 µM propidium iodide (Sigma-Aldrich) were added. The plate was maintained for 30 min at 37 °C. Images were analyzed in EVOS Microscope. The presence of living cells was evaluated by staining with calcein in comparison with the control and positive control.

### Scanning electron microscopy (SEM)

A scanning electron microscope (Zeiss EVO MA10) from Scanning Electron Microscopy Laboratory was utilized to determine pore structures of scaffolds. Samples were sputter coated with a layer of gold (Au) for observation at 10 kV at various levels of magnification (30x, 100x, 400x, 800x, 1600x). The surfaces of the scaffold were examined to identify any differences in pore size.

### Antibiogram

To verify the antimicrobial potential of the different scaffold formulations at a concentration of 1 mg/mL, a sensitivity test was performed for multiresistant bacteria of clinical relevance including ampicillin-resistant *Staphylococcus aureus* (MRSA), *Acinetobacter baumani* and *Klebsiella pneumoniae carbapenemase* (KPC). For the positive control, 10 μL of gentamicin (50 mg/mL) was used and 10 μL of PBS for the negative control.

First, the bacteria were inoculated in Brain Heart Infusion – BHI (Kasvi) medium and kept at 37 °C for 24 h. The inoculum were transferred to Tryptone Soy Agar – TSA (Kasvi) plates and kept at 37 °C for 24 h. The colonies were incubated in Tryptic Soy Broth – TSB (Kasvi) at 37 °C until they reached a turbidity of 0.5 on the McFarland scale and seeding was performed on the plate. Next, scaffolds and controls were placed and kept at 37 °C for 24 h and analyzed. The formation of a halo was observed around the scaffolds containing the bioactive compounds with antibiotic properties.

### In vivo tests

In vivo tests were performed according to Galiano et al. (2004), with modifications. Rubberized and flexible polymer disks were used to suture the edges of the excisional wounds in order to avoid their closure by the contraction process. The contraction is largely responsible for the closure of wounds in rodents, thus simulating the healing of injuries by secondary intention, in which primary approximation of the edges is not possible and healing occurs by re-epithelialization.

Three formulations of interest for the treatment of wounds were selected: CCh, CChNE and ChNE, only at a concentration of 1 mg/mL. C57BL/6 mice aged between 7 and 8 weeks were grouped as control group (excisional wounds with silicone disks), treated group 1 (CCh), treated group 2 (CChNE), treated group 3 (ChNE). Each group contained 16 animals.

The evolution of the healing process was evaluated macroscopically by measuring the area of the wounds, with the aid of a digital caliper, starting immediately after wound induction and occurring at predetermined intervals (1, 3 and 7 days) [20, 21]. Measurements were utilized to calculate the percentage of wound closure and perform healing kinetics.

### Histological analysis

On the 7th day after wound induction, the animals were euthanized. For histological and biochemical analyses, the wound region, along with the surrounding skin, was collected with the aid of an 8 mm circular biopsy punch.

To assess the formation of granulation tissue, a histological analysis was performed on the 7th day using a score to evaluate inflammation and epithelialization ranging from 0 to 3, with 0 indicating no inflammation/intact skin, 1 signifying discreet inflammation with the presence of few cells inflammatory cells and 1/3 of the epithelium present, 2 moderate inflammation with many inflammatory cells and more than 1/3 of the epithelium formed, and 3 severe inflammation with exaggerated presence of inflammatory cells and complete epithelium.

### Activity of pro-inflammatory enzymes

The remaining wound fragments were stored at −80 °C and subsequently weighed and processed to quantify the activity of the enzymes N-acetyl-β-glucosaminidase (NAG) and myeloperoxidase (MPO), for indirect evaluation of the infiltrate of macrophages and neutrophils.

To quantify the NAG activity, the samples were homogenized in 1 ml of 0.9% NaCl solution containing 0.1% Triton X-100 (Promega) and centrifuged at 960 × g, for 10 min at 4 °C. Subsequently, 150 mL of the supernatant of each sample was added to previously identified microtubes, containing 150 mL of citrate/phosphate buffer pH 4.5. In a 96-well plate, 100 µL volumes of samples were added in duplicate, in which 100 µL of the substrate (p-nitrophenyl-n-acetyl-β-D-glucosaminide – Sigma) was added at 2.43 nM, also diluted in citrate/phosphate buffer pH 4.5. Samples were incubated for 30 min at 37 °C. Then, at the end of the incubation period, 100 μL of 0.2 M glycine buffer pH 10.6 was added. Samples were measured at the absorbance of 400 nm. The results were analyzed to verify whether there was a difference in the inflammatory process between the samples.

Samples for quantification of MPO activity were homogenized in 1 mL of sodium phosphate buffer pH 5.4. The obtained supernatant (300 μL) was replaced with 600 μL of hexadecyltrimethylammonium bromide (HTAB, Sigma) 0.5% w/v diluted in phosphate buffer pH 5.4. Next, the samples were sonicated for 20 sec and subjected to 3 cycles of rapid freezing in liquid nitrogen and heating in a water bath. Subsequently, they were centrifuged for 10 min at 10,000 × g. For the reaction, 200 μL of sample and 100 μL of 3,3’-5,5’-tetramethylbenzidine (TMB) solution (Sigma-Aldrich, St. Louis) at 6.4 mM dissolved in dimethylsulfoxide (DMSO) were used with 100 μL of 2.4 mM H_2_O_2_ and diluted in phosphate buffer. The reaction was stopped with the addition of 100 μL of 4 M sulfuric acid. The activity of the MPO enzyme was determined by a spectrophotometer at the absorbance of 450 nm.

### Statistical analysis

Statistical analysis was performed using a computational program with the statistical package GraphPad Prism Version 9.0 (GraphPad Software, Inc., USA). The data were submitted to the Kolmogorov-Smirnov normality test. The variables - cell viability, wound closure, quantification of MPO and NAG enzymes and the average number of vessels/mm² - were analyzed using the One-way ANOVA multiple comparison test, followed by Dunnett’s post-test. For the pore size variable, the Kruskal Wallis post-test was used. Data were considered significant for *p* < 0.05.

## Results

### Fabrication and characterization of scaffolds

**Fig. 1:**
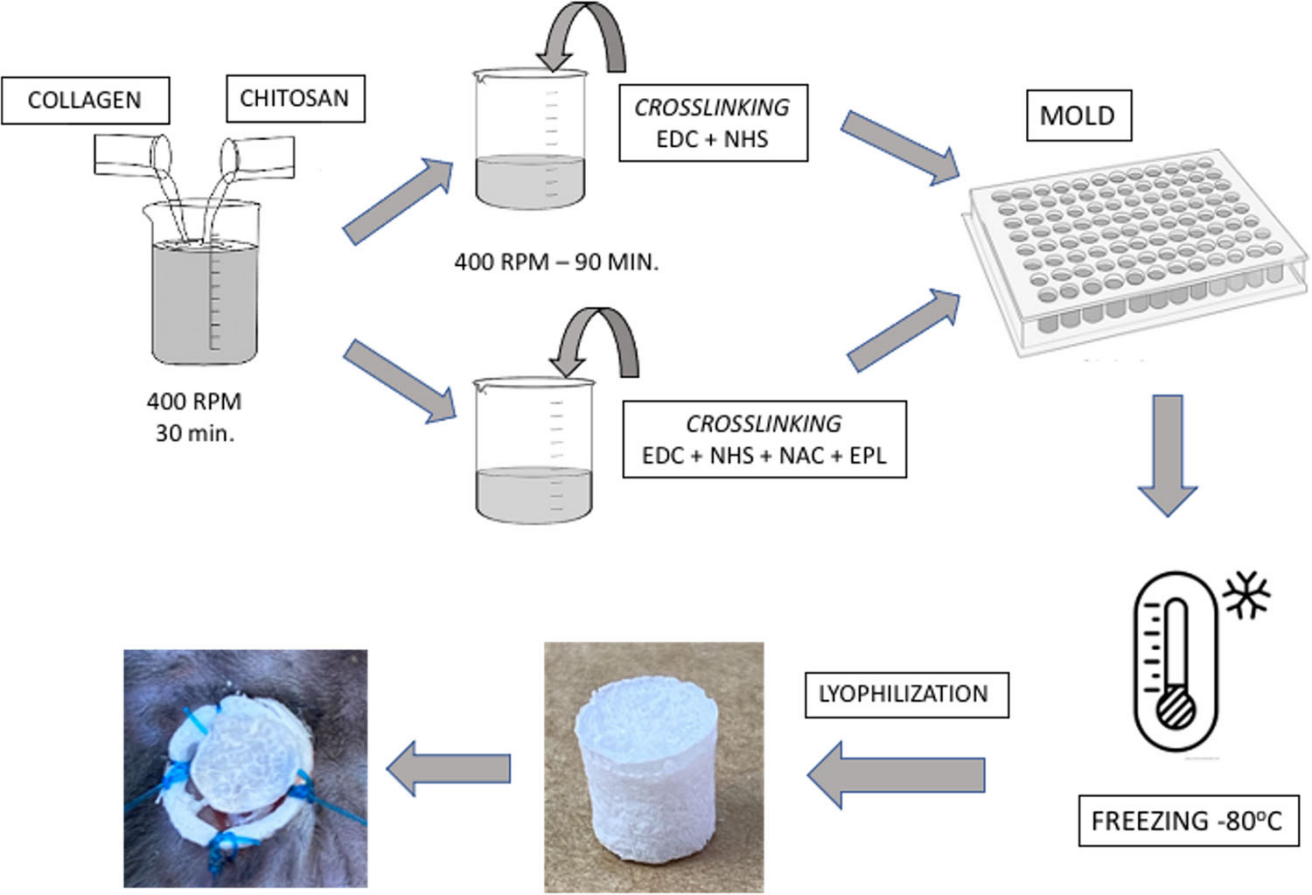
Representative scheme of the preparation of formulated scaffolds and real sample

In this research six different formulations were tested for comparison (C, CNE, CCh, CChNE, Ch and ChNE). The detailed process of preparing solutions, crosslinking, molding and lyophilization steps, as well as the appearance of the final product on the animal, are illustrated in Fig. 1.

The formulations C, CNE and Ch were considered the respective controls for the formulations CCh, ChNE and CChNE.

### Effect of scaffold extracts on HaCaT viability

**Fig. 2:**
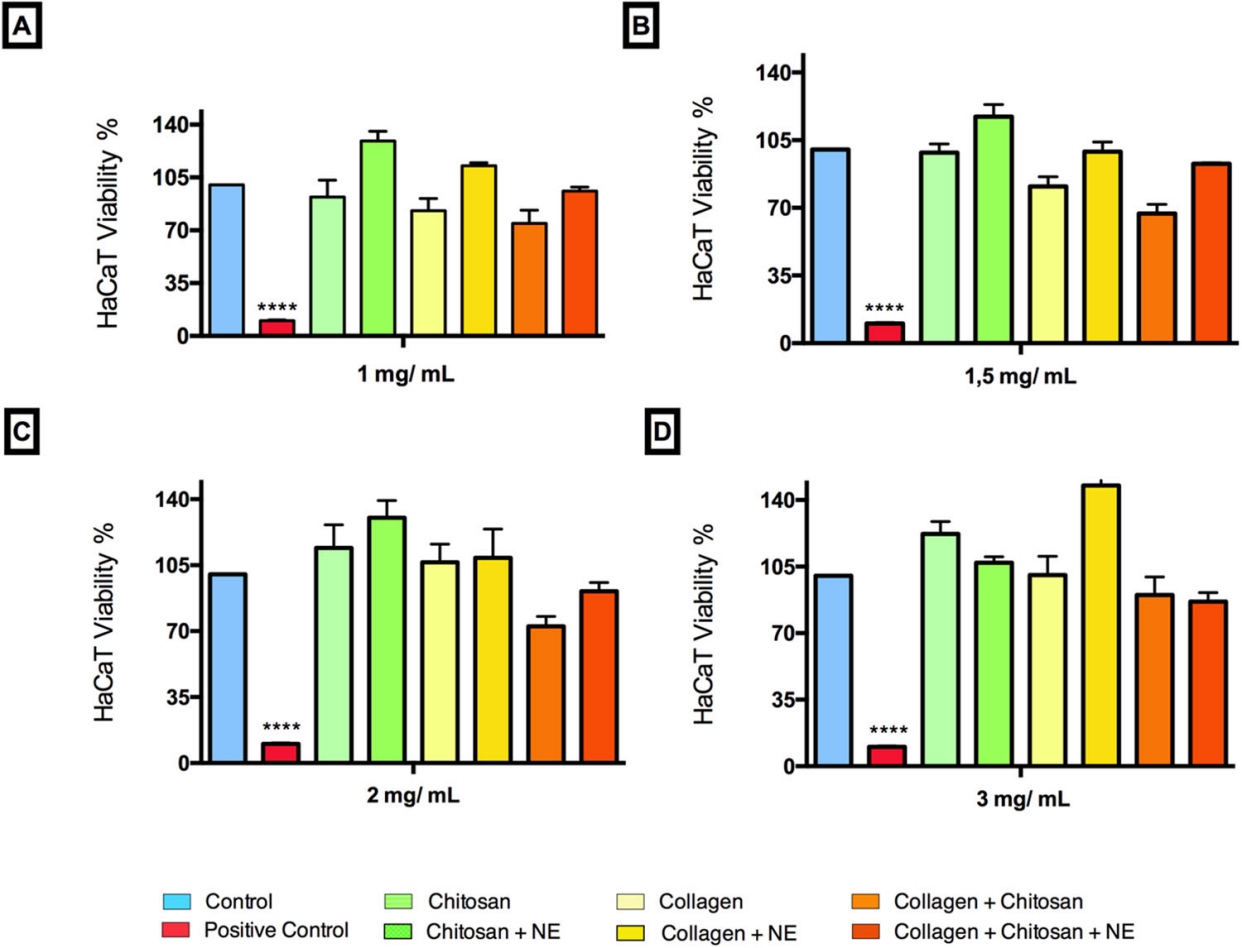
Cell viability of scaffolds extracts. **A** 1 mg/ mL solution scaffolds **B** 1.5 mg/ mL solution scaffolds **C** 2 mg/ mL solution scaffolds **D** 3 mg/ mL solution scaffolds. Controls: untreated cells, treated with 5% DMSO. X-axis values represent concentrations in μg/mL. * Statistically significant difference compared to negative control. Ordinary one-way ANOVA with multiple comparisons and Dunnet´s post-test were used. Data were considered significant when *p* value was less than 0.05

First, a screening assay was carried out applying the different formulation extracts of the collagenous sponge at concentrations of 1, 1.5, 2 and 3 mg/mL on human keratinocytes cell line (HaCaT), which resulted in high viability (over 80%) for all formulations containing collagen and chitosan (Fig. 2), except for CCh 1.5 mg/ mL formulation extract (62%). Based on this screening result, the 1 mg/ mL solution scaffold was chosen for the following assays.

### Live-dead assay

**Fig. 3:**
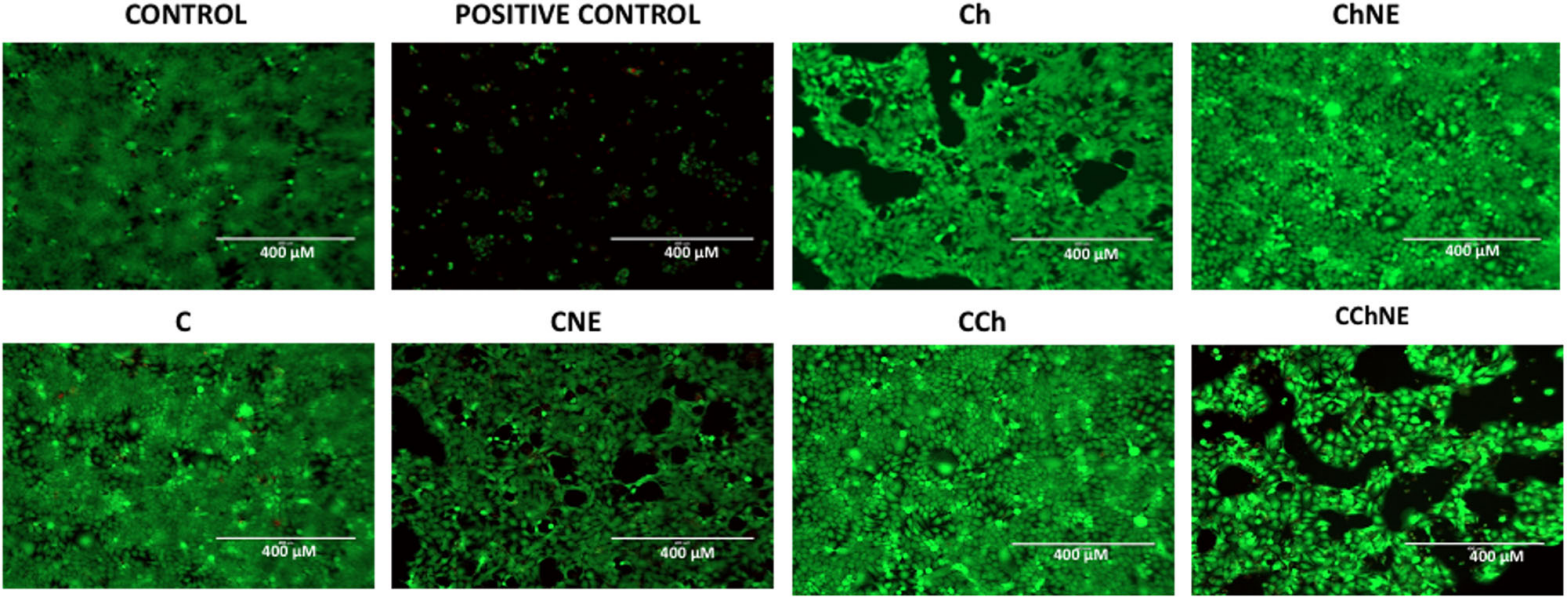
Live cells and cell death induced by 1 mg/ mL solution scaffolds extracts. Calcein, a PI overlay, was used for all images. Control: untreated cells. Positive control: cells treated with 5% DMSO. NE: NAC - ε-PL Magnification 100x. Scale bar: 100 μm

Cell viability results were also assessed by live-dead assay using calcein (viable cell marker) and PI (a cell death marker that permeates into cells with impaired cell membrane integrity) in HaCaT treated with 1 mg/ mL formulation extracts. Calcein staining showed that cells treated with C, CCh and Ch were viable and presented total confluence, whereas the formulations CNE, Ch and CChNE presented lower confluence, but were not labeled as dead cells. In the positive control for cell death, only cells in an intermediate state (not yet released from plastic) were stained, and the remaining cells were released and washed during the medium removal process for staining (Fig. 3).

### SEM morphology

**Fig. 4:**
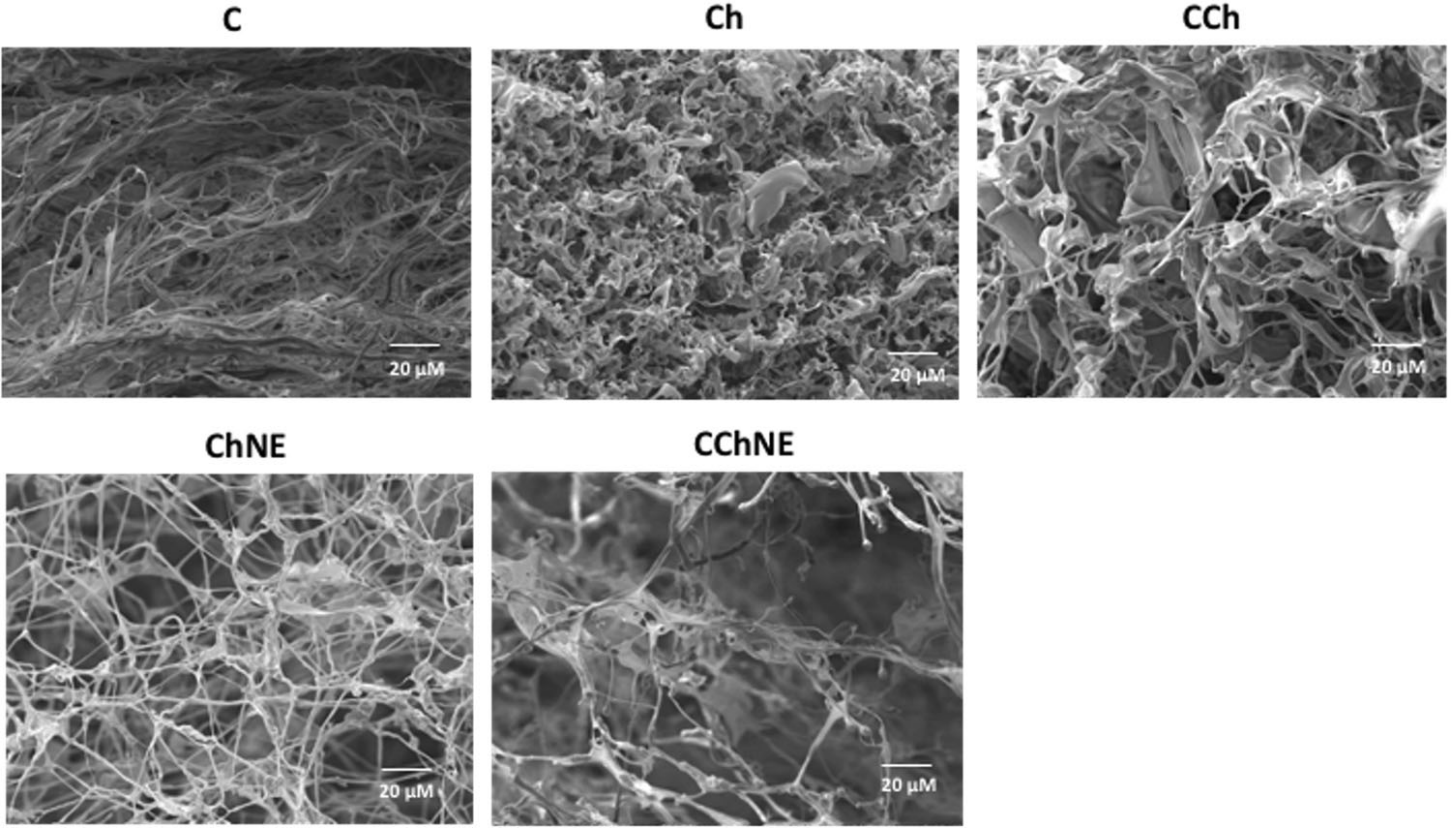
Morphology and ultrastructure of porous scaffolds. C: Collagen; CNE: Collagen - NAC - ε-PL; CCh: Collagen – Chitosan; CChNE: Collagen - Chitosan - NAC - ε-PL; Ch: Chitosan; ChNE Chitosan - NAC - ε-PL. Magnification: 800x. Scale bar: 20 μm

**Fig. 5:**
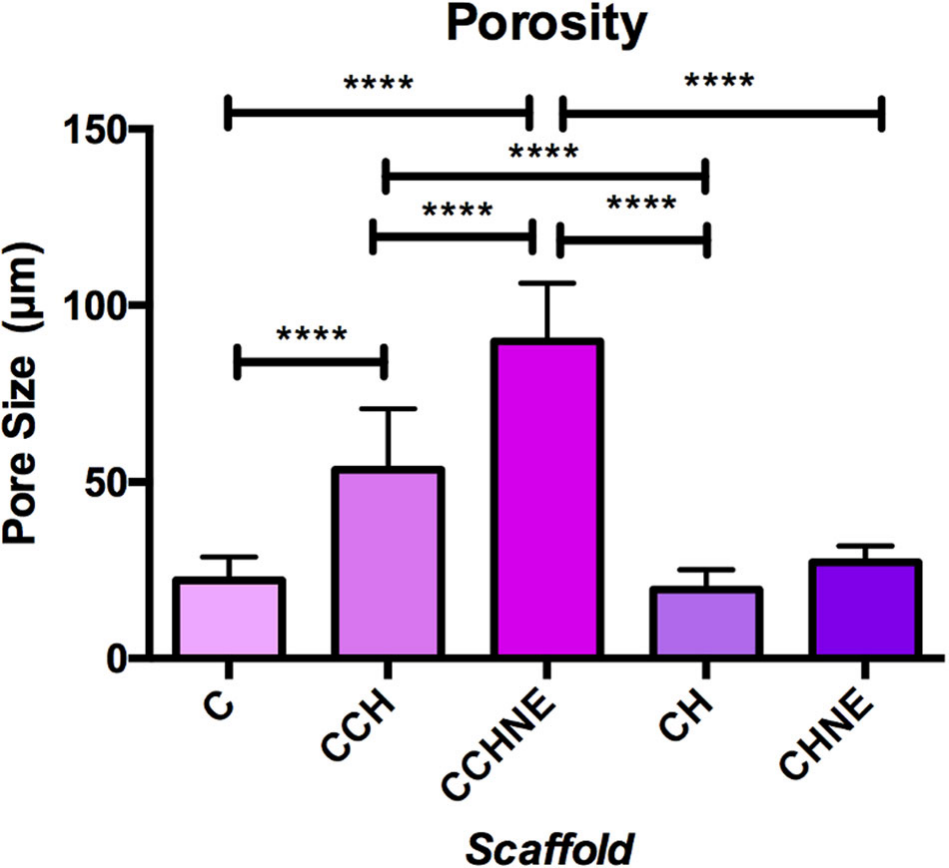
Evaluation of pore size before and after crosslink with chitosan. C: Collagen; CNE: Collagen - NAC - ε-PL; CCh: Collagen – Chitosan; CChNE: Collagen - Chitosan - NAC - ε-PL; Ch: Chitosan; ChNE Chitosan - NAC - ε-PL. 800x magnification. * Statistically significant difference in relation to the pure scaffold of each material. One-way ANOVA test with multiple comparisons and Kruskal Wallis post-test were used. Statistically significant difference *p* < 0.05

SEM analysis revealed that collagen-chitosan crosslinking increased the material porosity in the samples CCh and CChNE (Fig. 4). However, the morphology of the scaffolds was not homogeneous and showed variations of pore size. The mean pore sizes were C - 58.475 μm (± 22.1), CCh - 96.108 μm, (±53.5), CChNE - 104.854 μm (± 89.8), Ch - 56.925 μm (± 19.4) and ChNE - 66.9 μm (± 27.2) (Fig. 5).

### Antibacterial action

**Fig. 6:**
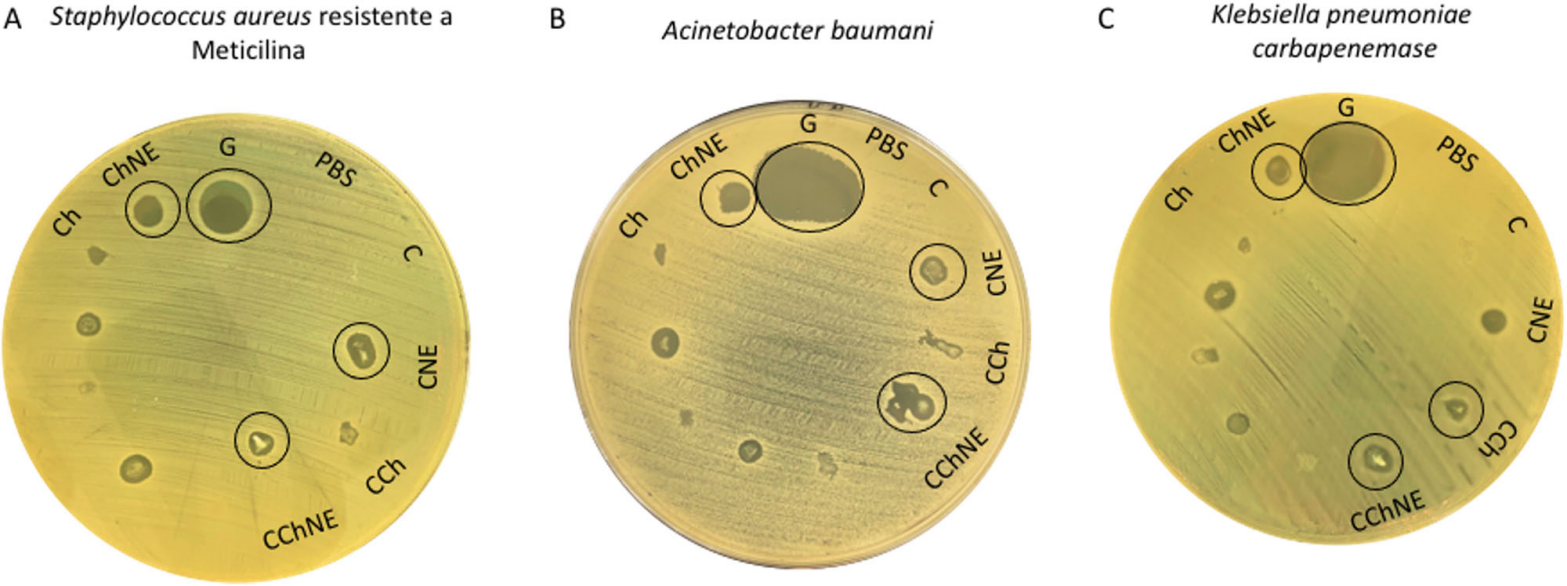
Antibacterial effect of scaffolds containing NAC - ε-PL. **A** Meticilin-resistant *Staphylococcus aureus* **B** *Acinetobacter baumani* **C** *Klebsiella pneumoniae carbapenemase*. Circles indicates the halo formation. G: Gentamicin; PBS: Phosphate Buffer Saline; C: Collagen; CNE: Collagen - NAC - ε-PL; CCh: Collagen – Chitosan; CChNE: Collagen - Chitosan - NAC - ε-PL; Ch: Chitosan; ChNE Chitosan - NAC - ε-PL

Scaffolds containing NAC and ε-PL (CNE, CChNE and ChNE) demonstrated antibacterial action against multiresistant bacteria of clinical relevance such as KPC, MRSA and *Acinetobacter b*. This result can be observed in Fig. 6 by the presence of a halo around the scaffolds with this property, while in the other formulations (C, CCh and Ch) the effect was not detected.

### Wound closure analysis

The experimental model of excisional wounds used a stabilizing ring to avoid the muscle contraction characteristic of mouse skin. The scaffolds for treatment were chosen after the in vitro analyses according to the interest for this research; therefore, CCh, CChNE and ChNE were selected.

**Fig. 7:**
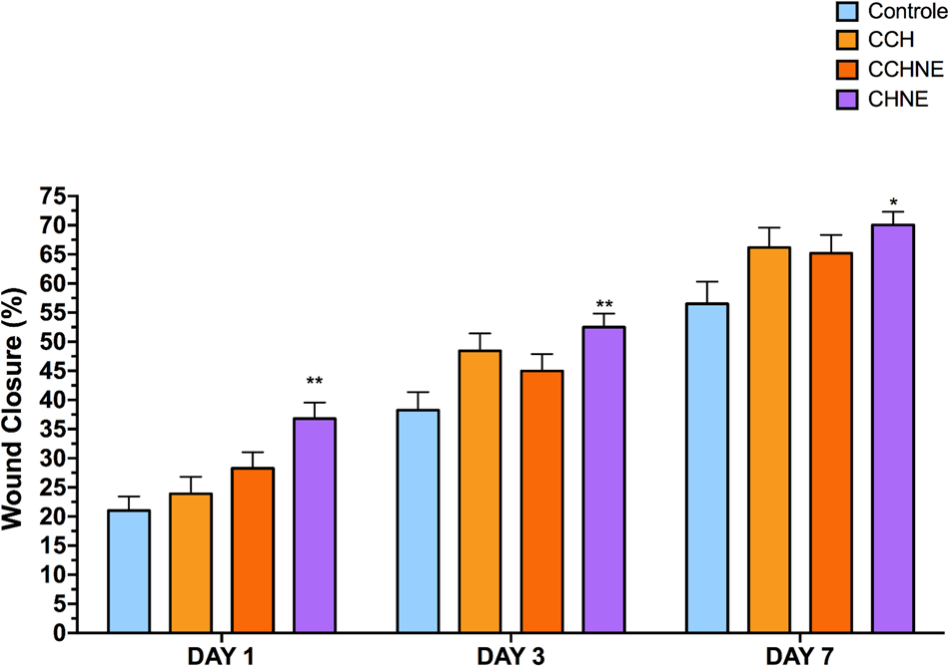
Wound closure on days 1, 3 and 7. CCh: Collagen – Chitosan; CChNE: Collagen - Chitosan - NAC - ε -PL; Ch: Chitosan; ChNE Chitosan - NAC - ε -PL. *Statistically significant difference compared to the control group. One-way ANOVA test with multiple comparisons and Dunnet’s post-test were used. Data were considered significant when the value of *p* < 0.05

In the analysis of wound closure, the ChNE formulation showed a statistically significant difference when compared to the control group on days 1, 3 and 7. At the end of the treatment, the group treated with ChNE presented 70% wound closure. The other groups of the CCh and CChNE formulations did not differ significantly, as shown in Fig. 7.

**Fig. 8:**
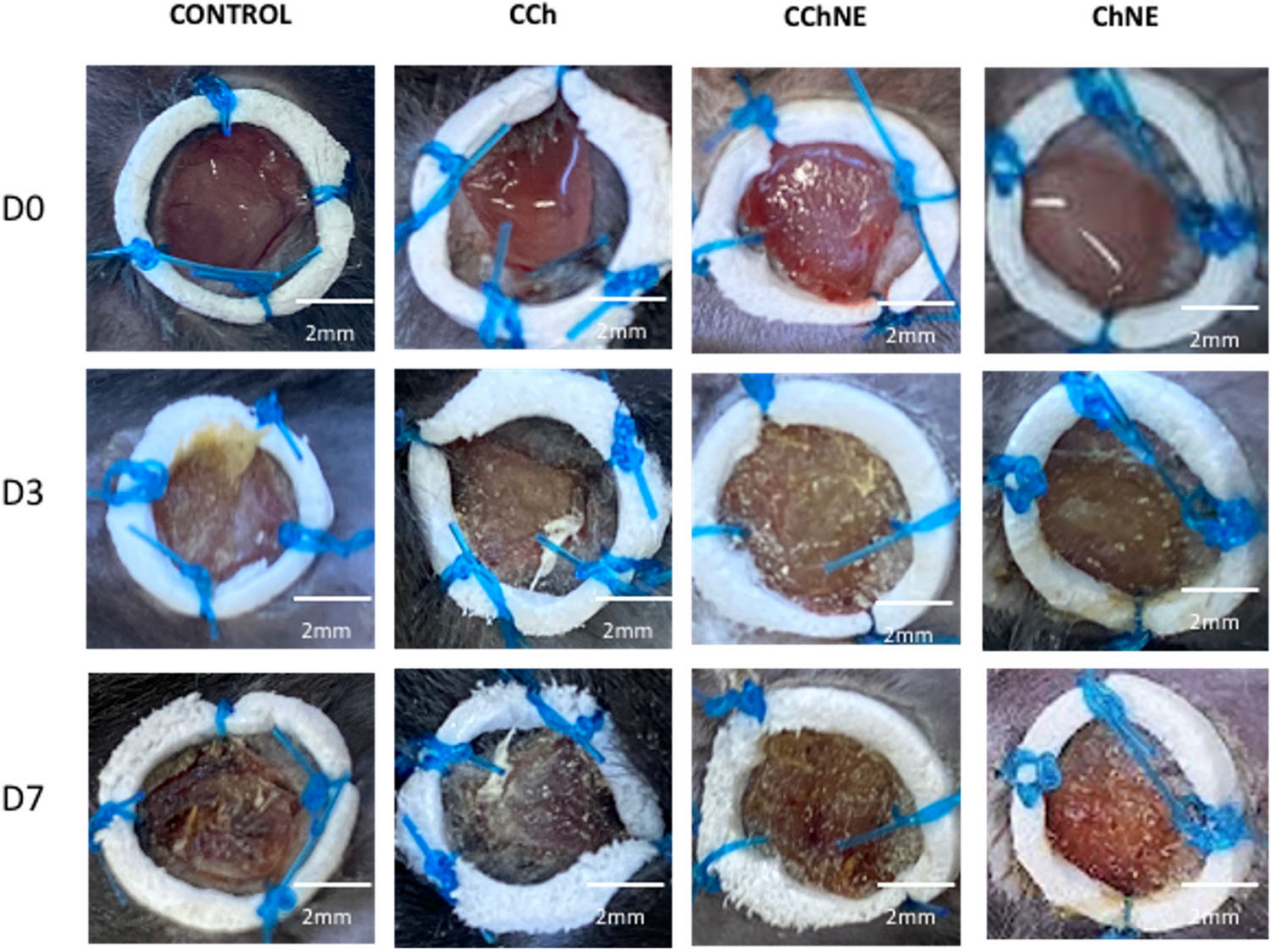
Representative images of wound closure on days 0 (before scaffolding), and at days 3 and 7. CCH: Collagen – Chitosan; CChNE: Collagen - Chitosan - NAC - ε -PL; Ch: Chitosan; ChNE Chitosan - NAC - ε -PL

The scaffolds were progressively absorbed, revealing fragments in the wound bed within 24 h and complete absorption in 72 h. The presence of low-secretion granulation tissue was observed throughout the treatment. No macroscopic signs of hemorrhage, tissue necrosis or wound infection, were observed at the end of the experiment (Fig. 8). The animals displayed behavioral patterns compatible with good general health.

### Assessment of inflammatory infiltrate and epithelialization

**Fig. 9:**
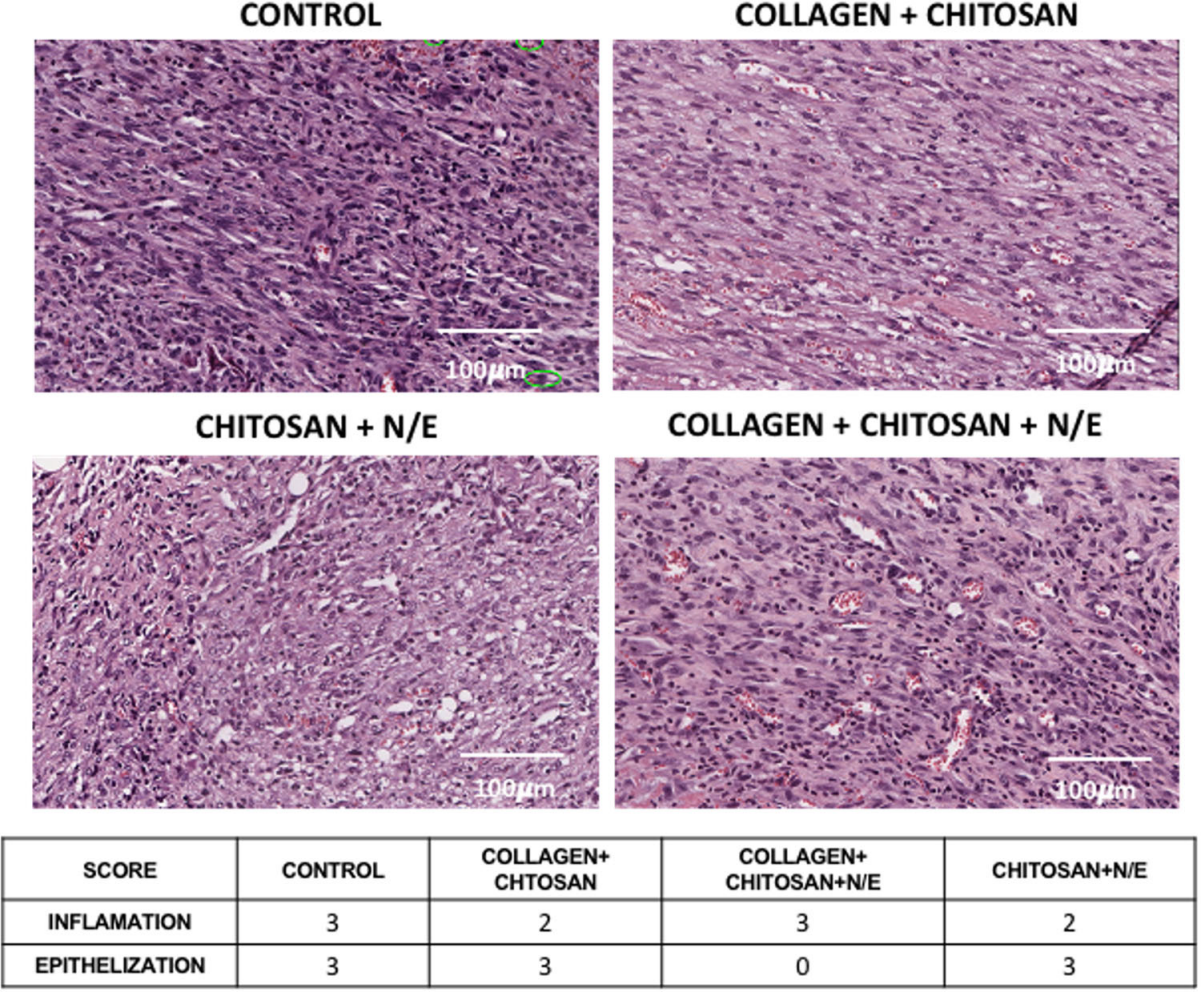
Images representing the inflammatory infiltrate of the wounds **A** and epithelialization **B**. CCh: Collagen – Chitosan; CChNE: Collagen - Chitosan - NAC - $\underline{\varepsilon }$ -PL; Ch: Chitosan; ChNE Chitosan - NAC - $\underline{\varepsilon }$ -PL. 20x magnification using ImageScope software

A moderate inflammatory infiltrate was observed in the treatments with the CCh and ChNE formulations (score 2) with presence of inflammatory cells that was discreet at the border of the wound, and moderate in the central region, indicating the healing process from edges to center, evidenced by a loosely organized extracellular matrix. The control groups and other treatment showed severe inflammation, evidenced by the exacerbated presence of inflammatory cells throughout the whole wound extension (Fig. 9). Epithelialization was complete (score 3) for CCh and ChNE and partial (<1/3) for CChNE (1). The presence of scaffold was not detected at the histological level in any of the treated groups, indicating complete absorption 7 days after treatment.

### Evaluation of the activity of pro-inflammatory enzymes

**Fig. 10:**
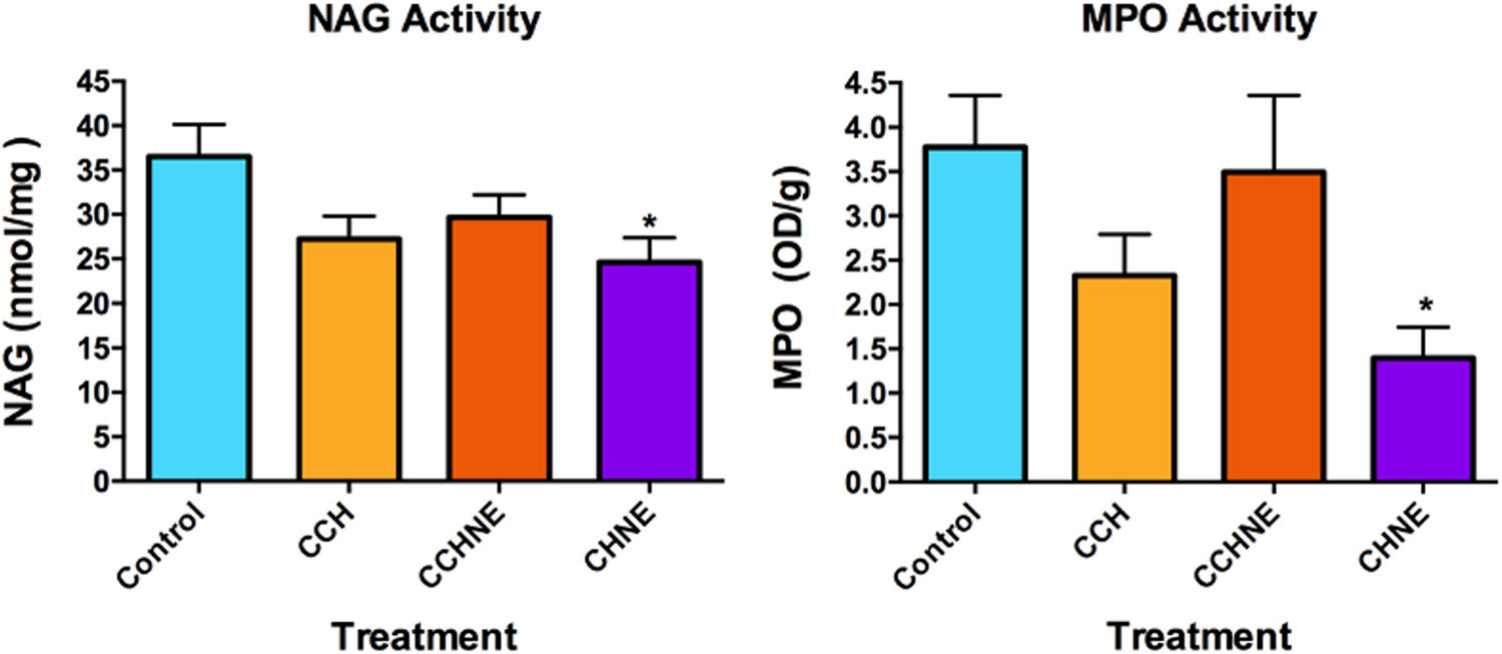
Quantification of NAG and MPO activity. CCh: Collagen – Chitosan; CChNE: Collagen - Chitosan - NAC - $\underline{\varepsilon }$ -PL; ChNE Chitosan - NAC - $\underline{\varepsilon }$ -PL. *Statistically significant difference compared to the control group. One-way ANOVA test with multiple comparisons and Dunnet’s post-test were used. Data were considered significant when *p* value < 0.05

The activities of NAG and MPO enzymes were evaluated to determine the respective activities of macrophages and neutrophils in the wounds. A significant reduction of NAG and MPO was observed in the lesions treated with the ChNE (24.6 NAG nmol/mg; 1.3 MPO OD/g) formulations when compared to the control group (36.5 NAG nmol/mg; 3.7 MPO OD/g). The other treatments did not show statistically significant results (Fig. 10).

## Discussion

The wound healing process is complex, requiring the presence of several inflammatory cells, chemokines, cytokines and nutrients at the wound site. It consists of three stages: the inflammatory, the proliferative and the remodeling stage, which may suffer environmental or pathological modifications, resulting in undesirable effects such as infection and chronic inflammation [22]. To avoid such effects, wound care is a crucial step that is directly linked to the associated morbidity and the effective resolution of healing. The ideal dressing provides adequate supplies of oxygen, moisture, angiogenesis and nutrition, in addition to protecting against pathogens and trauma [22, 23].

In this context, the development of wound dressings that provide the appropriate conditions to accelerate the healing process or prevent chronicity has been subject of research. Traditional dressings with dry gauze can delay the process or traumatize the area when removed. Biopolymers are being researched as targets for regenerative medicine, assisting in the development of scaffolds that can contain bioactive substances, nutrients or growth factors [24–26].

Given the higher rate of collagen degradation over synthesis in chronic wounds, considering exogenous collagen supplementation at the wound site emerges as a viable option [25, 27]. Therefore, animal collagen-based biomaterials have been adapted for the manufacture of scaffolds to create an environment that mimics the extracellular matrix in chronic wounds [1, 5]. Also, chitosan exerts hemostatic action in contact with traumatic wounds through platelet adhesion and promotes erythrocyte agglutination. It also induces macrophage activation and has antibacterial and fungicidal action [7, 8]. Thus, chitosan scaffolds bioconjugated with collagen have been studied for application in tissue regeneration, due to biocompatibility and porosity that can promote cell proliferation [25, 28]. In light of all the evidence suggesting the benefits of collagen-chitosan bioconjugates, we proposed the development of a collagen scaffold bioconjugated with chitosan and antibacterial compounds such as NAC and ε−PL. We hypothesized that such a scaffold would demonstrate good wound regeneration capabilities.

In resazurin and live/dead assays there was an increase of cell viability in scaffold solutions. Cell proliferation was greater when treated with collagen associated with chitosan and cell morphology was preserved. Corroborating these results, a study with hydrogel-conjugated collagen and chitosan formulation showed positive effects on the proliferation of L929 cells and human mesenchymal stem cells [18]. In this study the proportion of dead cells in total was extremely low, and the cells presented typical morphology. There were more living cells attached to the chitosan-collagen and chitosan-NAC/ε−PL than other formulations. Another research study using marine collagen-chitosan cryogels has also reported non-cytotoxic (80%) behavior by L929 cells, confirmed by live/dead assay showing predominantly live cells [8].

While the resazurin and live/dead assays provided sufficient evidence of non-toxicity formulations, our assessment of the scaffolds’ ultrastructure aimed at selecting non-cytotoxic and porous materials for further in vivo assays. We demonstrated that the collagen scaffold bioconjugated with chitosan has greater porosity, being suitable for tissue repair [28]. As expected, the results found in the SEM demonstrate the ultrastructure of the different formulations, showing that samples of pure collagen and chitosan (C, Ch) presented higher density. Scaffolds containing collagen and chitosan (CCh, CChNE) displayed complete and larger pore structures. Corroborating these results, Kafi et al. [29] demonstrated that collagen and chitosan scaffolds presented higher porosity and a more homogeneous structure when compared to controls. The authors report that scaffolds with greater porosity provide higher cell proliferation, demonstrating that bioconjugation of matrices have physical properties superior to pure collagen. Other research studies [8, 19] found the same result, in which structures containing collagen-chitosan crosslinking have larger pores than pure materials. However, one study by Chao Deng et al. [18] observed larger pores in collagen materials and smaller and more homogeneous pores with the addition of chitosan, producing a denser structure, in contrast to what was found not only in the current study but also in prior ones. Despite our consistent aim to enhance porosity in our formulations, purportedly to achieve increased cell proliferation and viability as suggested by literature, our study revealed an unexpected outcome. It demonstrated that despite lower porosity, scaffolds with bioconjugation of chitosan with NAC and E-PL supported higher cell viability in both in vitro and in vivo assays. This suggests that the addition of NAC and ε−PL to the scaffold may confer greater benefits to cells than solely increasing porosity.

In addition to non-cytotoxic and porous structures, we were also looking for a biomaterial with bactericidal properties, so we add NAC and ε-PL to the formulations and verify their action. Bacterial infection is a serious complication in the treatment of chronic wounds, which can form antibiotic-resistant biofilms. To prevent this complication, dressings with antibacterial substances are a desirable option [30]. The antibacterial action of the NAC and ε-PL additives was confirmed by the bacterial sensitivity test, but a more complete antibiogram assay is needed for quantitative evaluation. Shivakumar et al. [9], report that the use of nanocomposite collagen dressings with chitosan inhibit not only microbial infections, but also the activity of wound matrix metalloproteinases by inflammatory cells that are present in excessive amounts in chronic wounds. Furthermore, the authors revealed that the proposed collagen-chitosan dermal substrate aids both vascularization and cell proliferation of the tissue. In a study conducted by Mayandi et al. [31], ε-PL was used as a bioactive compound in wound dressings and demonstrated efficacy in reducing bacterial load and promoted tissue healing with better results than conventional dressings. In another study, hydrogels with a porous structure based on chitosan bioconjugated with polyvinylalcohol and ε-PL, showed excellent results in healing and antibacterial action against *E. coli* and *S. aureus* [30].

After confirming the bactericidal action, we also evaluated the anti-inflammatory action of the scaffolds to verify the potential of the bioactive compounds. In the inflammatory phase of healing, neutrophils act to decontaminate the lesion. However, in chronic wounds they can cause damage by producing free radicals and oxygen, resulting in oxidative stress, thus slowing the healing process due to excessive amounts of reactive oxygen species (ROS) found in chronic wounds [32]. Our results showed a reduction of inflammatory cells in the wounds treated with collagen hydrogel bioconjugated with chitosan, NAC and ε-PL when compared to the control group. In this context, n-acetylcysteine acts as an important bioactive anti-oxidant in chronic wounds, as observed by Li et al. [33], as well as Ozkaya et al. [34], who carried out dermal regeneration experiments in mice, and also demonstrated a reduction in the tissue oxidative stress during the healing process. The collagen/chitosan compound gel developed by Li et al. [33], showed good results in wound healing with increased healing rate and shorter duration than other treatments, almost complete after 14 days. The compound increased granulation tissue, collagen deposition and wound vascularity, and was also able to inhibit the growth of *S. aureus*. In another study conducted by Tsai et al. [35], the use of NAC for treating burns in an experimental rat model, demonstrated that NAC promotes wound healing and accelerates re-epithelialization. In addition, NAC induced collagenous expression of MMP-1, which is important in the process of tissue repair and remodeling. The current research has verified that the expected effects of the chitosan+ NAC/ε-PL combinations were similar to those reported in the literature. The formulation of chitosan and NAC/ε-PL scaffolds surprisingly exhibited greater antibacterial properties and accelerated the wound healing process compared to treatments involving collagen bioconjugation [36].

Despite our initial presumption that collagenous scaffolds combined with chitosan would perform better, our findings revealed that chitosan combined with NAC and ε-PL yielded superior results in terms of both cell viability and wound closure in the model used. The observed wound closure and decrease in inflammatory cells within the ChNE group indicate the promising potential of this scaffold for tissue regeneration.

## Conclusion

In this study, we sought to obtain a new scaffold formulation with biomimetic, antioxidant, antibacterial and porous properties for application in dermal regeneration of chronic wounds. The desired properties were verified by characterization methods and in vivo biological analysis, and showed desirable characteristics for the adequate treatment of chronic wounds, by promoting biocompatibility, antibacterial action, antioxidant properties and a porous framework for cell proliferation. In our future studies, we aim to conduct research on diabetic and infected wounds to assess the effectiveness of the scaffolds in vivo using these models.
